# Soluble gC1qR in Blood and Body Fluids: Examination in a Pancreatic Cancer Patient Cohort

**Published:** 2015-09-03

**Authors:** Ellinor IB Peerschke, Ricardo JMGE Brandwijk, Francine R Dembitzer, Yayoi Kinoshita, Berhane Ghebrehiwet

**Affiliations:** 1 Department of Laboratory Medicine, Memorial Sloan Kettering Cancer Center and Department of Laboratory Medicine and Pathology, Weill Cornell Medical Center, NY, NY, USA; 2 Hycult Biotech, R&D Department, Uden, The Netherlands; 3 Department of Pathology, Mount Sinai School of Medicine, NY, NY, USA; 4 Department of Medicine, Stony Brook University, Stony Brook NY, USA

**Keywords:** gC1qR, Blood, Pancreatic cancer, Peritoneal fluid, Complement, ELISA

## Abstract

**Background:**

gC1qR is a multifunctional cellular protein that has been linked to inflammation and cancer. gC1qR is highly upregulated in adenocarcinomas as compared to normal tissue counterparts, and soluble gC1qR (sgC1qR) has been detected *in vitro* in the pericellular milieu of proliferating malignant cells.

**Aim:**

The present study explored the tissue expression of gC1qR in pancreatic cancer by immunohistochemistry, and the presence of sgC1qR *in vivo*, by examining blood and malignant effusions from patients with metastatic pancreatic adenocarcinoma.

**Methods:**

Tissue expression of gC1qR by pancreatic adenocarcinoma was visualized by immunohistochemistry. SgC1qR was quantified in serum from healthy volunteers (n=20) and pancreatic cancer patients (n=34), as well as in malignant pleural (n=23) and peritoneal effusions (n=27), using a newly developed, sensitive immunocapture sandwich ELISA.

**Results:**

Overexpression of gC1qR was confirmed in pancreatic adenocarcinoma compared to nonmalignant pancreatic tissue. Moreover, increased serum levels of sgC1qR (0.29 ± 0.22 ng/ml) were noted in patients with metastatic pancreatic cancer compared to healthy controls (0.15 ± 0.10 ng/ml) (mean ± S.D.) (p=0.035). In 11 of 16 patients for whom sequential samples were available, serum sgC1qR levels rose with disease progression, and paralleled changes in tumor biomarkers, CEA and CA19.9. In addition to blood, sgC1qR was detected in malignant pleural (0.55 ± 0.47 ng/ml) and peritoneal effusions (0.57 ± 0.38 ng/ml).

**Conclusion:**

This study provides the first evidence for the presence of sgC1qR *in vivo*. The ability to detect sgC1qR in blood and body fluids will enable further studies to elucidate its pathophysiology in malignancy.

## Introduction

gC1qR is a multifunctional cellular protein of 33 kDa that has been implicated in inflammation [1] and malignancy [2]. It was initially identified as a receptor for the globular heads of C1q [3–5]. gC1qR is localized in several cellular compartments, including mitochondria [6–9], endoplasmic reticulum and nucleus, as well as the cell surface, where it appears to be associated with lipid rafts [5,10–15].

The ability of gC1qR to modulate the activity of ligands both inside and outside of the cell is increasingly recognized. Several binding partners have been identified, including C1q [3,16], vitronectin [17], and high molecular weight kininogen [18–20]. These interactions lead to classical complement pathway activation with generation of inflammatory cytokines [21,22], and activation of the kinin system with production of bradykinin and ensuing vascular permeability [22]. gC1qR is also important for the maintenance of oxidative phosphorylation [6], and stable knockdown of gC1qR has been shown to shift metabolism from oxidative phosphorylation to glycolysis [23]. In addition, gC1qR has been implicated as a potential regulator of cell proliferation, adhesion, migration, and invasion [15,24].

We hypothesize that gC1qR may play a role in carcinogenesis. Indeed, differential gC1qR expression has been reported in a variety of tumors, particularly epithelial tumors compared to their nonmalignant histologic counterparts [25,26]. gC1qR expression on the cell membrane has been shown to promote tumor cell migration and invasion *in vitro* [27], and recent evidence suggests that gC1qR overexpression in primary ovarian carcinomas is associated with decreased overall survival and decreased progression free survival [28]. The observation that increased gC1qR expression occurs also in benign inflammatory and proliferative lesions [26], suggests that increased gC1qR expression may be a marker of cell proliferation, with potential diagnostic and therapeutic applications.

In addition to cellular gC1qR expression, soluble gC1qR (sgC1qR) has been observed *in vitro* [21]. sgC1qR may exert potent physiologic effects including activation of complement, coagulation, and kinin systems. Moreover, sgC1qR was shown to bind to cultured endothelial cells and to induce the bradykinin/B1 receptor [29], which promotes angiogenensis [30].

The presence of sgC1qR *in vivo* has not been established. The present study developed a reliable, high-sensitivity ELISA to screen blood and body fluids as proof of principle for the existence of sgC1qR *in vivo*. Serum samples from healthy volunteers and patients with pancreatic cancer were tested. In addition, malignant pleural and peritoneal effusions were evaluated. The data provide clear evidence of the presence of sgC1qR in blood and malignant body fluids, and suggest that sgC1qR levels may change with disease progression. The ability to quantify sgC1qR levels will enable further research into gC1qR biology in health and disease.

## Methods

### Biological samples

Histologic sections of pancreatic adenocarcinoma were obtained from the archives of the Department of Pathology at Mount Sinai School of Medicine, New York, NY, according to Institutional Review Board-approved protocols. Immunohistochemical analysis was performed on archived paraffin-embedded tissue sections (4m thick), as previously described [26].

Residual patient serum (n=34) as well as peritoneal (n=27) and pleural (n=23) fluid samples were obtained from the Clinical Laboratories at Memorial Sloan Kettering Cancer Center (MSKCC), New York, NY, after all diagnostic testing had been completed. Serum from healthy volunteers was provided from the Clinical Laboratory serum repository. Samples of serum and malignant pleural or peritoneal effusions were from patients with advanced (Stage IV) pancreatic cancer. All blood and body fluid samples were de-identified before inclusion in the study. This study was approved by the MSKCC Institutional Review Board for the Protection of Human Subjects.

### sgC1qR analysis

sgC1qR was detected with a new *in vitro* assay developed for the quantitative determination of sgC1qR in plasma, serum, culture supernatants, and body fluids (Hycult Biotech, Uden, The Netherlands). The assay was performed according to manufacturer instructions. Briefly, ELISA plates/wells (Nunc, Thermo Scientific, Netherlands) coated with anti-gC1qR antibody (HM2014) and blocked for nonspecific binding with bovine serum albumin were washed, and incubated with recombinant gC1qR standards or samples, diluted in dilution buffer (PBS/0.1%BSA), for 1 hour at room temperature. After 4 washes, the wells were reacted with a biotinylated polyclonal anti-gC1qR antibody in dilution buffer for 1 hour at room temperature. After washing, the conjugate was allowed to react with streptavidin-conjugated Horse Radish Peroxidase (HRP). Color development was performed by addition of Tetramethylbenzidine (TMB) substrate. The reaction was stopped using an acidic buffer. The absorbance at 450nm was measured with a microplate reader. sgC1qR quantitation was achieved by comparing the cleavage of the TMB substrate by patient or control samples relative to a set of recombinant gC1qR standards ranging in concentration from 0–20 ng/ml.

### Measurement of cancer biomarkers, CEA and CA19.9

Serum CEA and CA19.9 levels were determined in the Clinical Biochemistry Laboratory at MSKCC using a fluorometric enzyme immunoassay on the Tosoh AIA 2000 (Tosoh USA, Grove City, OH), as part of the patient’s clinical evaluation. Reference ranges for CEA (0–5 ng/ml) and CA19.9 (0–40 ng/ml) were established by the performing laboratory.

### Data analysis

Mean and standard deviation were calculated for individual data sets. Statistical significance between data sets was analyzed by Mann-Whitney U test for unpaired data, or Student t-test for paired data. Significance was defined as p<0.05.

## Results

**Figure 1:**
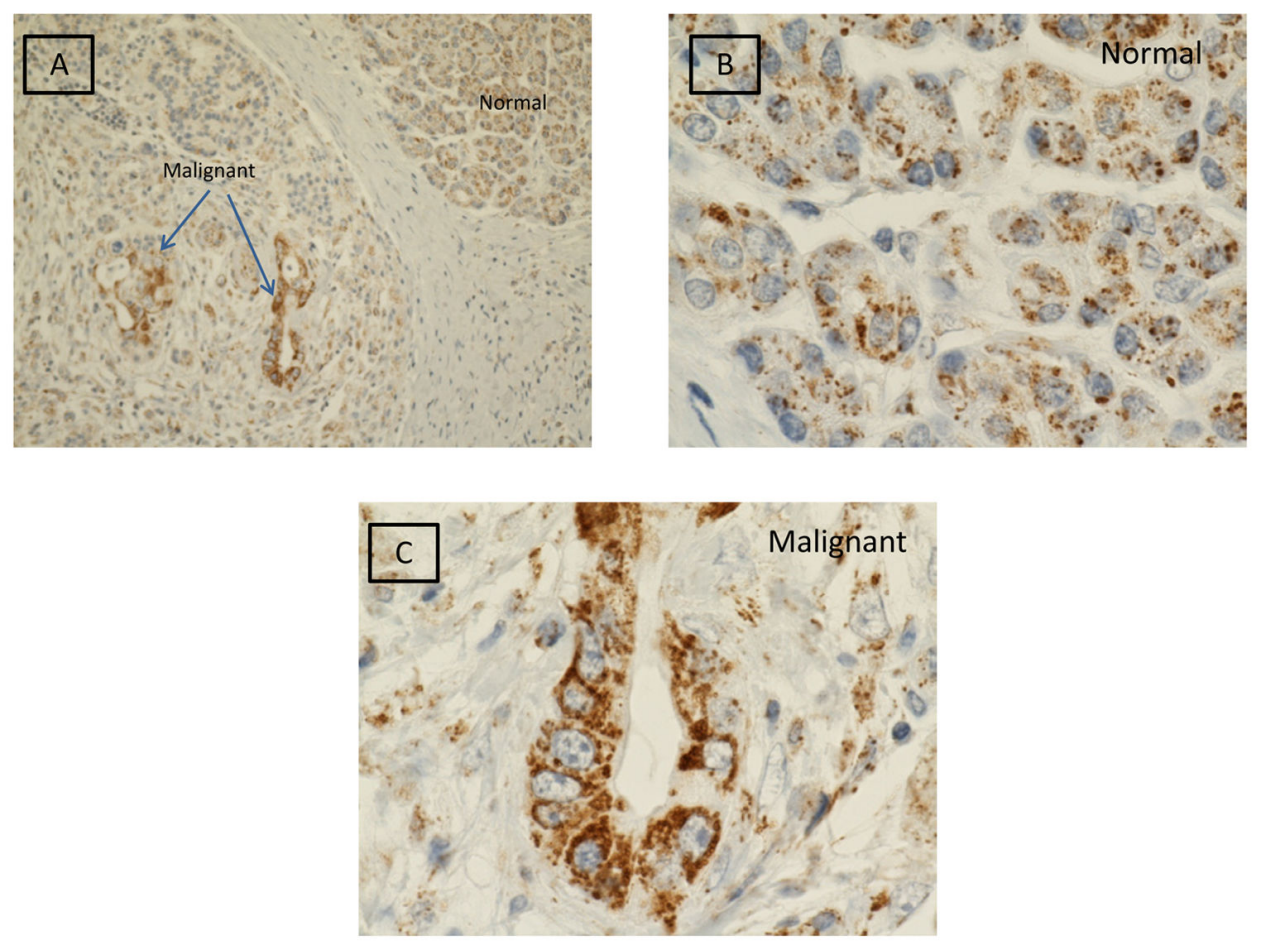
Immunohistochemical staining of gC1qR (brown stain) in malignant and normal cells of the exocrine pancreas. Light microscopic images obtained at low power (20x) (Panel A) and high power (100x oil immersion) (Panels B and C) are shown.

Figure 1 illustrates gC1qR expression in malignant and benign pancreatic tissue. Marked gC1qR expression is noted in malignant cells. Relative to benign cells, dense cytoplasmic gC1qR inclusions are seen by light microscopy in malignant cells of the exocrine pancreas. Both low (20x) and high (100x oil immersion) magnification views are shown.

### sgC1qR ELISA

**Figure 2:**
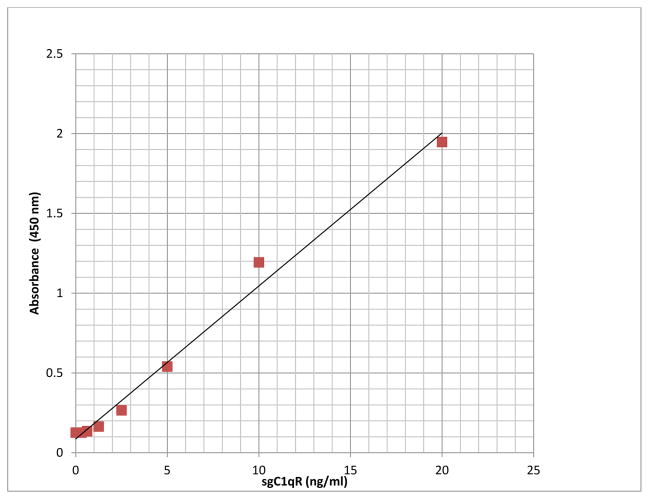
Representative results of sgC1qR ELISA standard curve showing linear response between absorbance and sgC1qR concentration from 0–20 ng/ml.

A newly developed sandwich ELISA assay was used to measure sgC1qR. The optimum combination of the capture (murine monoclonal antibody) and detection antibody (polyclonal rabbit anti-human gC1qR) was reached based on most optimal signal/noise ratio (data not shown). No signals were obtained in absence of capture or detection antibody (data not shown). The assay was linear between 0–20 ng/ml. Over this concentration range, the relationship between absorbance and sgC1qR concentration yielded a correlation coefficient of 0.99 (Figure 2). Using serum samples or normal plasma, anticoagulated with either EDTA or sodium citrate (blood collection tubes from Becton Dickinson, Franklin Lakes, NJ), to which known concentrations of recombinant gC1qR were added (spiked samples), gC1qR recovery was >95% with an intra- assay variability of 17 + 3%. Anticoagulation of blood with heparin produced false elevations in gC1qR quantitation and is therefore not recommended (data not shown). To minimize matrix effects, plasma/serum and body fluid samples were diluted 1:20 in the assay.

### sgC1qR levels in serum from healthy volunteers and patients with pancreatic cancer

**Figure 3:**
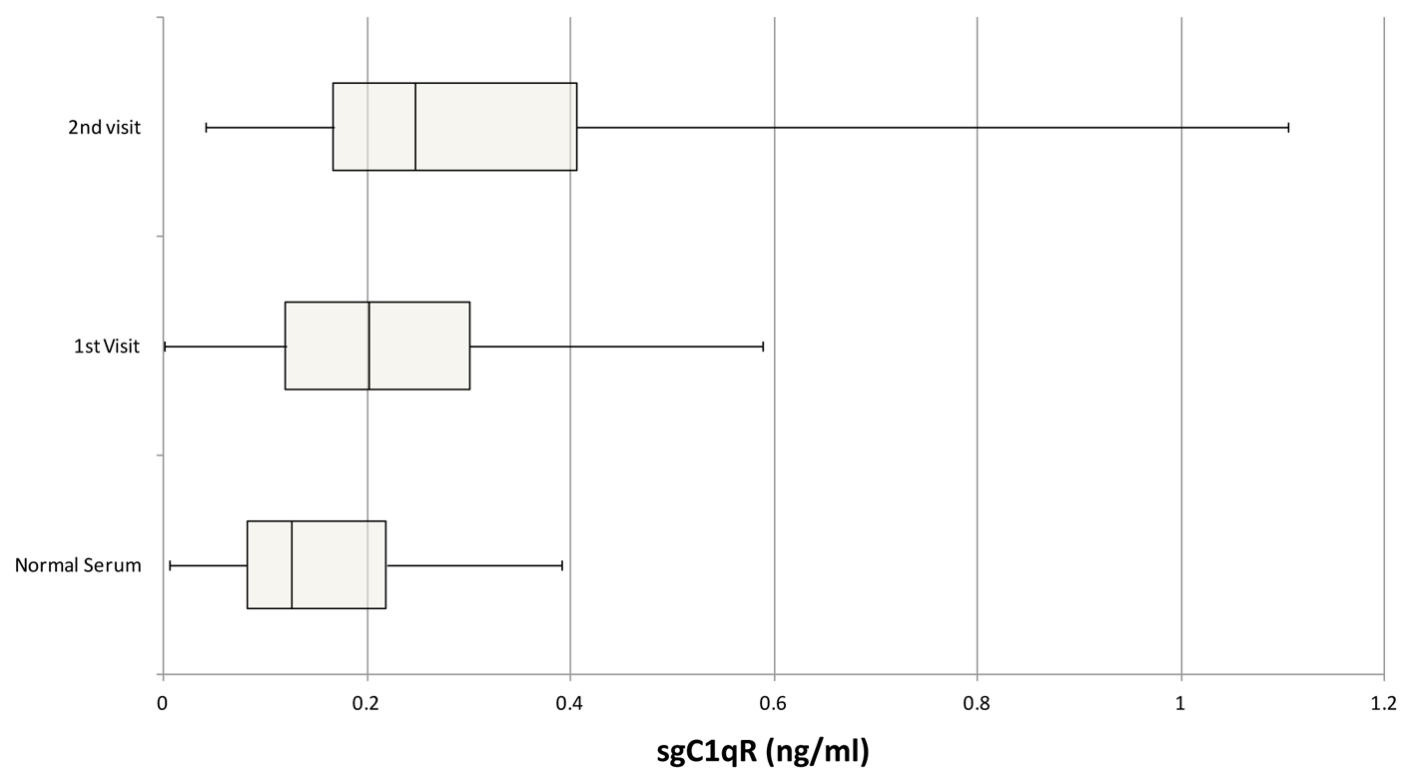
Comparison of sgC1qR in normal serum and serum from patients with pancreatic cancer obtained at two different visits 3–12 months apart, designated visit 1 and 2. Data are presented as box and whisker plots showing median sgC1qR levels and the interquartile range, representing the middle 50% of sgC1qR levels, framed by the box. The error bars (whiskers) represent sgC1qR values in the upper (25%) and lower (25%) quartiles.

Serum samples for healthy volunteers and patients with stage IV pancreatic cancer were tested for sgC1qR. Patients (n=34) (male=19, female =15) ranged in age from 40–83 years. Healthy volunteers (n=20) (male=10, female =10) ranged in age from 35–70 years. sgC1qR was detected in serum of healthy volunteers, and increased levels (p =0.035) were found in patients with pancreatic cancer, although significant overlap was appreciated (Figure 3). Further increases in sgC1qR levels were noted with disease progression (p=0.005), in samples from patients obtained during a second visit, 3–12 months later.

To further evaluate the relationship between sgC1qR levels and progression of disease, we examined changes in the relationship between sgC1qR and tumor markers CA19.9 and CEA using serum sample pairs obtained 3–12 months apart. Although direct correlations between gC1qR and CA19.9 or CEA were not statistically significant, (r= 0.1056 and 0.1692, respectively), the relationship between changes (increase/decrease) over time between sgC1qR and tumor markers showed agreement for 75% of serum samples (12 of 16 serum sample pairs). Increases in sgC1qR levels, CEA, and CA19.9 levels were seen in 11 of 16 samples over time, whereas decreases in sgC1qR, CEA, and CA19.9 were noted for one serum pair. In the remaining 25% of sample pairs (4 of 16), tumor markers increased and sgC1qR levels decreased. The reason for the discordance is currently unknown.

### sgC1qR in malignant effusions

sgC1qR levels were measurable also in malignant pleural and peritoneal paracentesis fluids (Table 1). Benign effusions were not available for comparison. Interestingly, sgC1qR levels appeared to be higher in malignant effusions than in serum.

## Discussion

Inflammation, complement, and cancer are tightly linked, with complement playing both antagonistic and supportive roles in carcinogenesis [2]. gC1qR has been reported to contribute to inflammation by direct activation of the complement, kinin, and coagulation cascades [1]. Moreover, overexpression of gC1qR has been demonstrated in adenocarcinomas and in a range of epithelial cancers [25,26]. Data from several sources suggest a role for gC1qR as a potential therapeutic and diagnostic target [28,31–34]. For example, in a mouse model, changes in cellular gC1qR expression levels appear to coincide with specific stages of tumor progression [32], and in a study of 63 patients with breast cancer, gC1qR mRNA expression levels correlated with axillary lymph node metastasis and poor patient survival [34]. Similar observations were reported in patients with ovarian cancer, in whom gC1qR overexpression correlated with poor outcome [28].

Since sgC1qR has been reported *in vitro* in culture supernatants [21], and may play a role in angiogenesis and modulation of inflammation [1,2], the present study used a newly developed quantitative assay to measure sgC1qR in blood and body fluids. These results provide proof of principle for the presence of quantifiable sgC1qR *in vivo*.

Measurable sgC1qR levels were detected in the serum of healthy volunteers, and increased levels were found in the serum of patients with pancreatic cancer. Interestingly, changes in patient sgC1qR levels were observed with progression of disease, many of which paralleled changes in known tumor markers, such as CEA and CA19.9. In addition, sgC1qR was detected in malignant pleural and peritoneal effusions. Although the source of sgC1qR is not identified, these findings would support secretion of sgC1qR from cancer cells, as observed in *in vitro* studies [21]. However, sgC1qR secretion from inflammatory cells or apoptotic cells cannot be ruled out.

Whereas the current study provides proof of principle for the presence of sgC1qR *in vivo* in blood and body fluids, further studies are required to characterize sgC1qR expression in patients with cancer, and to understand its pathobiology. Limitations of the present study relate to the small sample size, and an inability to evaluate benign/infectious or inflammatory blood and body fluids. In addition, patient and control groups matched for comorbidities are required to determine the specificity of sgC1qR as a marker of malignancy or inflammation, particularly as *in vitro* evidence suggests gC1qR upregulation by inflammatory cytokines [35]. Results of the present study encourage further investigation of sgC1qR *in vitro* and *in vivo*. Since gC1qR antibodies used in the ELISA cross react with mouse and rat gC1qR, it is conceivable that the assay may be adapted for studies of sgC1qR in rodent models.

## Figures and Tables

**Table 1:** Summary of sgC1qR levels in serum and body fluids of patients with pancreatic cancer

Specimen Type	n	sgC1qR (ng/ml) (mean ± S.D.)
Normal Serum	20	0.15 ± 0.10
Patient Serum	34	0.29 ± 0.22
Pleural Fluid	23	0.55 ± 0.44
Peritoneal Fluid	27	0.57 ± 0.38
